# Supplementary material for: Metabolic Responses to an Acute Glucose Challenge: The Differential Effects of Eight Weeks of Almond vs. Cracker Consumption in Young Adults
Source: medRxiv. 2024 May 20:2024.05.19.24307571. Preprint. [Version 1] doi: 10.1101/2024.05.19.24307571 (PMC11142291; doi:10.1101/2024.05.19.24307571)
Supplement: Supplement 1 [file media-1.docx]

**Supplementary Figure 1**. Heat map of Pearson’s correlations of metabolite fasting and AUC 120 variables in a) the almond and b) cracker groups. In the extremes of the color gradient, red represents a strong positive correlation while blue represents a strong negative correlation. The size of the correlation represents the statistical significance i.e., big rectangle indicates P<0.05, small rectangles indicate P>0.05, missing rectangles indicate that correlations are between the same variables.


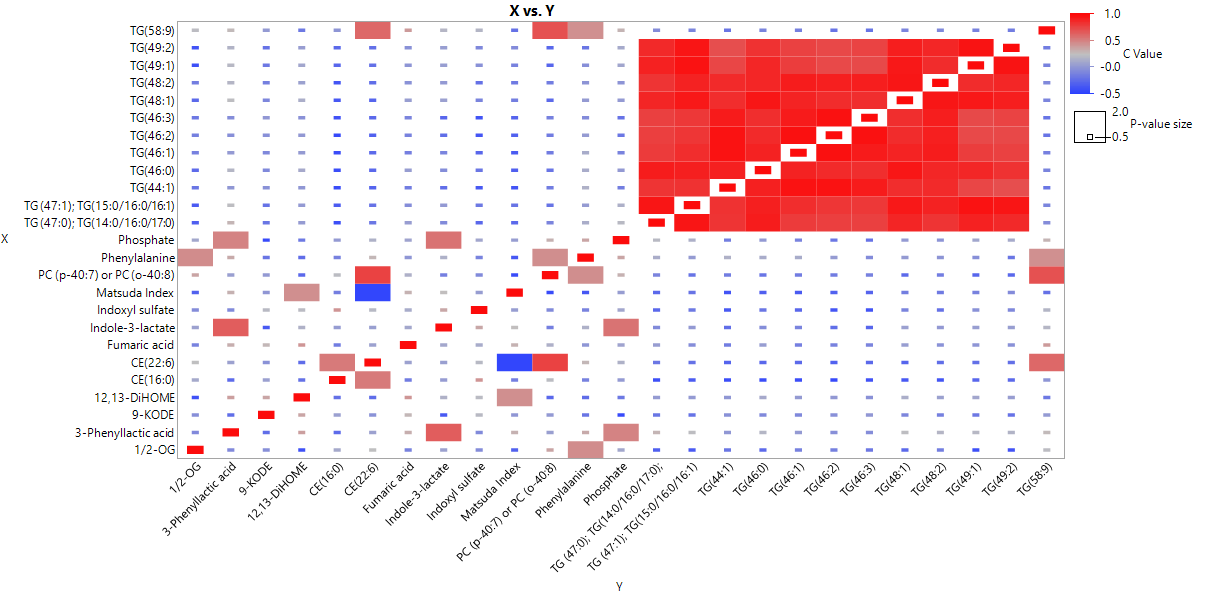


**A)**


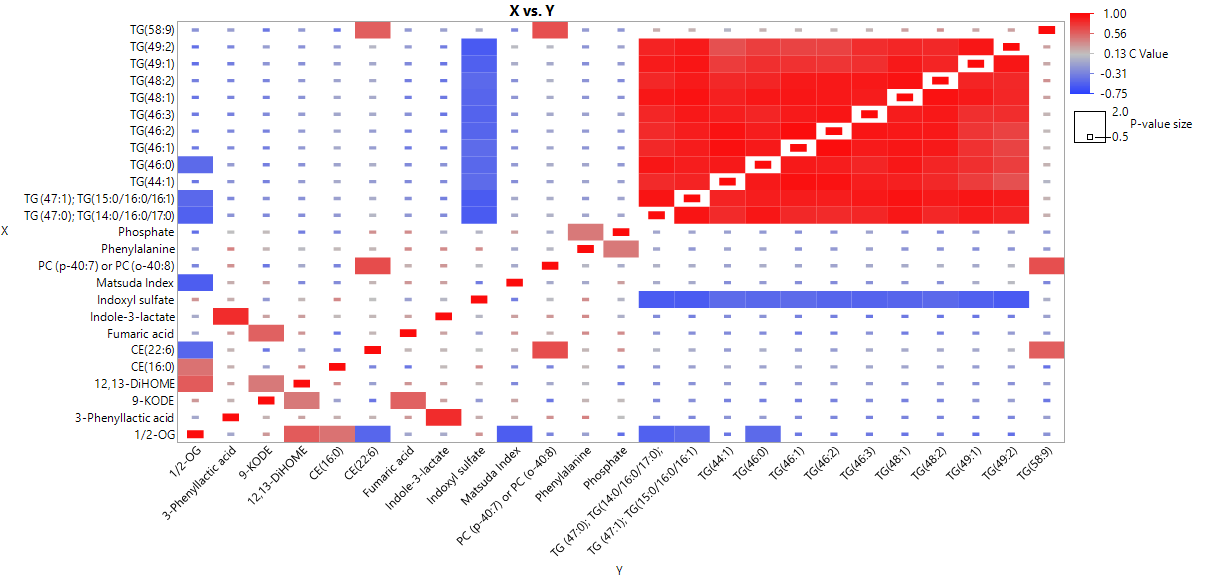


**B)**
